# Case Report: A rare pediatric case of hepatitis B virus infection with acute disseminated encephalomyelitis/Guillain–Barré syndrome spectrum diseases

**DOI:** 10.3389/fped.2025.1697707

**Published:** 2026-01-06

**Authors:** Yingxue Li, Dongxu Hu, Xing Shen, Xiaoyan Mao

**Affiliations:** 1 Department of Pediatrics, Children Hematological Oncology and Birth Defects Laboratory, The Affiliated Hospital of Southwest Medical University, Sichuan Clinical Research Center for Birth Defects, Luzhou, Sichuan, China; 2 Southwest Medical University, Luzhou, Sichuan, China

**Keywords:** ADEM+/GBS spectrum diseases, convulsions, hepatitis B virus, infectious diseases, neurological damage

## Abstract

A 12-year-old girl with convulsions was incidentally found to be infected with hepatitis B virus(HBV), with HBVdeoxyribonucleic acid (DNA) and HBsAg positivity in the cerebrospinal fluid. The patient experienced frequent convulsions and consciousness disorders, and positive anti-GQ1b/anti-GD1b antibodies were detected in the cerebrospinal fluid. Acute disseminated encephalomyelitis(ADEM)/Guillain–Barré syndrome (GBS) spectrum disorders were observed on brain and spinal cord magnetic resonance imaging, indicating a high likelihood of HBV-related ADEM+/GBS spectrum disorders. After treatment with intravenous hormone, immunoglobulin, antiviral therapy using entecavir, and antiepileptic medication, the neurological damage completely resolved, the hepatitis B viral load decreased significantly, and liver function returned to normal. ADEM+/GBS spectrum diseases are immune-mediated disorders of the central nervous system that occur after viral infection. However, the potential relationship between hepatitis B virus infection and ADEM+/GBS spectrum diseases remains an area of ongoing investigation. In this case, the combination of hormones and intravenous immunoglobulin did not affect the anti-HBV effect of entecavir, and the patient fully recovered from the neurological damage within three months of disease onset. This case highlights hepatitis B-related neurological diseases and encourages further sharing of clinical experiences.

## 1 Introduction

Hepatitis B virus (HBV) infection is a significant global health problem, affecting approximately 296 million people worldwide. HBVinfection can lead to liver-related diseases andvarious extrahepatic complications, such as HBV-associated nephritis. HBV can also cause neurological diseases; however, its pathophysiology remains unknown (1). About 1% of GBS cases have been found to be associated with acute hepatitis (A, B, C, D, and E) (2). In children, the detection of hepatitis B virus in the central nervous system is considerably rarer. We report the case of a child with HBVdeoxyribonucleic acid (DNA) and HBsAg-positive cerebrospinal fluid (CSF) who presented with convulsions as the initial manifestation.

## 2 Case report

A 12-year-old girl with intermittent convulsions and fever was admitted to our pediatric intensive care unit (11/24/2024). The convulsions presented as seizures, with a maximum body temperature of 38 °C and no symptoms of respiratory or digestive tract infections. Meanwhile, her mother stated that the patient had no history of infection or immunomodulatory exposure in the past eight weeks (such as gastrointestinal or respiratory infections, febrile illnesses, vaccinations, or other immune-modulating exposures). She had been healthy, denied exposure to toxins or drugs, and had a normal birth and growth history. The patient had received a hepatitis B immunoglobulin injection and vaccine after birth and was vaccinated according to the plan, but not tested for hepatitis B. The mother had contracted hepatitis B during pregnancy and was not treated. The motherhad no family history of epilepsy.

Physical examination at admission revealed a shallow coma, a Glasgow Coma Scale score of E1V1M4, positivity for the bilateral Babinski sign, and negativity for the Kernig sign. The results of other neurological and physical examinations were normal.

Routine peripheral blood parameters, inflammatory indicators, and liver function were normal. Plasma HBVDNA quantitative polymerase chain reaction (PCR) results were 6.88 × 10^7^ IU/mL, HBsAg > 50,000 IU/mL (reference range: 0–0.05 IU/mL), HBeAg > 250 PEIU/mL (reference range: 0–0.1 PEIU/mL), and HBcAb > 25 IU/mL (reference range: 0–0.35 IU/mL); whereasblood tests for HIVand hepatitis C werenegative.

The first CSF examination (11/24/2024) revealed a slightly elevated white blood count (WBC: 21 /μL, reference range: 0–15/μL), with 17% mononuclear cells, 83% multinucleated cells, no red blood cells, and normal sugar, chloride, and protein levels. Sequencing read counts were normalized using reads per million (RPM) to facilitate comparative analysis across samples with varying sequencing depths. The RPM value for each microbial species was calculated using the following formula: RPM = (Number of reads uniquely mapped to a pathogen/Total number of sequenced reads after quality control) × 1,000,000. CSF was tested for 150 targeted pathogens, and only HBV was positive (90 RPM, calculated from 235 reads out of 2.61 million total reads); no other bacteria, fungi, viruses, and parasites, including *Mycobacterium tuberculosis*, nontuberculous mycobacteria, mycoplasma, and chlamydia, were detected. Antibodies for autoimmune encephalitis in the CSF and peripheral blood of this patient were also negative. The second CSF examination (12/2/2024) revealed a nearly normal WBC, no red blood cells, and normal sugars, chlorides, and proteins. The second CSF was also tested for 150 targeted pathogens, and only HBV was positive, but the copies decreased (5 RPM, calculated from 17 reads out of 3.41 million total reads), HBsAg tested positive, and no other pathogens were found (Table 1).

**Table 1:** CARE Checklist of information to include when writing a case report.

Laboratory parameter	2024-11-24	2024-11-25	2024-11-27	2024-12-2	2024-12-3	2024-12-8	2024-12-9	Reference range
CSF Profile								
WBC cells/μL	21			11				0–15
RBC cells/μL	0			0				–
mononuclear cells %	17			–				–
Multiplenuclear cells %	83			–				–
Glucosemmol/L	4.15			3.57				2.8–4.2
Chloridemmol/L	122.5			125.2				111–123
Protein g/L	0.213			0.161				0.2–0.4
Pressure mmH_2_O	220			400				60–180
HBV-DNA reads	235			17				–
HBsAg				Positive				Negative
Plasma								
HBV DNA IU/mL		6.88*107					5.87*105	–
Plasma								
Alanine Aminotransferase U/L	26.2		16.4		25.8	40.4		7–30
Aspartate Aminotransferase U/L	24		22.7		42.6	41.4		14–44
Total Bilirubin μmol/L	13.9		7.9		8	3.7		0–23
Direct Bilirubin μmol/L	2.8		3.7		1.6	1.7		0–7
Indirect Bilirubin μmol/L	11.1		4.2		6.4	2		0–20
Total Protein g/L	63.8		66.1		74.6	57.8		65–84
Albumin g/L	41.6		36.1		31.7	29.2		39–54

**Figure 1:**
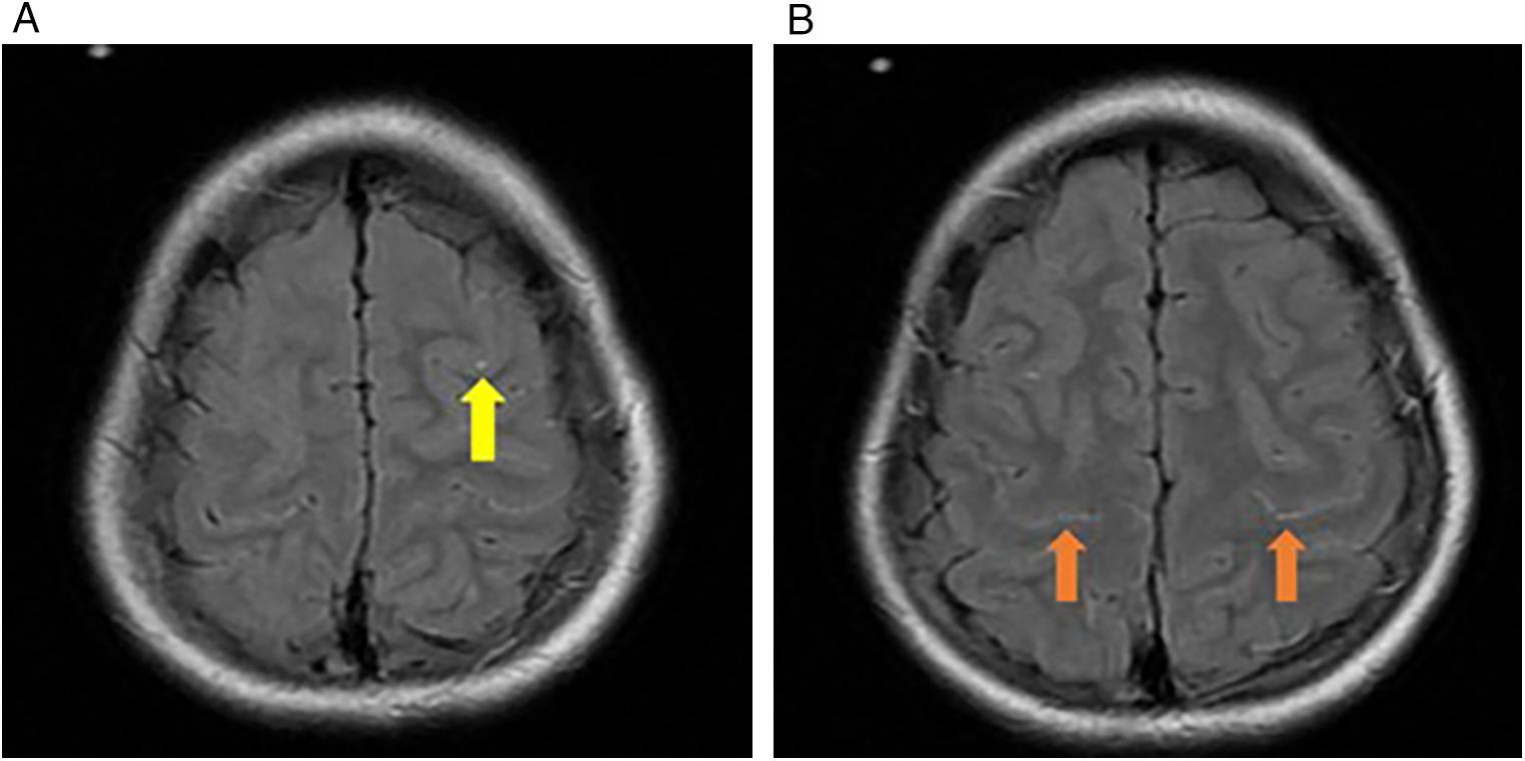
**(A)** punctate FLAIR slightly high, signal shadows observed in the frontal lobe sulci (yellow arrow); **(B)** linear FLAIR slightly high, signal shadows are observed in the bilateral parietal lobe sulci (orange arrows).

Magnetic resonance imaging (MRI) was performed on 11/26/2024 (Figure 1). Electroencephalography (EEG), performed on 12/7/2024, showed a continuous state of convulsion (bilateral occipital region),near-continuous high-amplitude slow waves in the bilateral frontal area, and a lack of sleep-wake cycles. Abdominal ultrasonography(11/28/2024) revealed a thickened and enhanced liver echo.

Owing to frequent convulsions and apnea, the patient was mechanically ventilated and received intravenous midazolam (2 μg/kg/min), levetiracetam (500 mg, q12h), and topiramate via a nasogastric tube (150 mg, q12h). To address the risk of viral encephalitis, the following treatments were administered: intravenous acyclovir (500 mg, q8h) and intramuscular interferon (50 μg, qd) for antiviral treatment; entecavir via a nasogastric tube (0.5 mg, qd) for oral anti-HBV treatment; intravenous low-dose dexamethasone (0.3 mg/kg/d); IVIG (total 1 g/kg) for immune regulation; and mannitol combined (125 mL, q6h) with glycerol fructose (250 mL, q12h) to lower intracranial pressure. After 2 weeks of treatment, plasma HBVDNA decreased significantly (5.87 × 10^5^ IU/mL), but intermittent convulsions and consciousness disorders persisted.

The patient was referred to another hospital. Routine CSF and biochemical tests were negative, as was the autoimmune encephalitis antibody test. CSF HBVDNA and antigen levels were not retested. Cerebral MRI(12/13/2024) showed cortical and subcortical abnormalities in both cerebral hemispheres, accompanied by cytotoxic edema in the brain tissue; thus, infection could not be ruled out. EEG (12/13/2024) showed mixed activity of 2–7.5 Hz throughout the brain in the sedation and eye-opening states; therefore, viral and autoimmune encephalitis could not be excluded. Dexamethasone was stopped, and intravenous methylprednisolone (2–3 mg/kg/d) was administered instead. Lacosamide was added, and levetiracetam and topiramate were administered as anticonvulsants. Entecavir (for anti-HBV treatment) was maintained, and thefrequency of convulsions decreased; however, the consciousness disorder persisted.

The patient was referred to a third hospital. The CSF showed anti-GQ1b/anti-GD1b antibody positivity, and cerebral MRI (12/23/2024) showed abnormal T2hyperintense signals depicted symmetrically in both cerebral hemispheres, affecting the cortex with restricted diffusion. The findings were nonspecific andindicated post-ictal changes, acute demyelinating encephalomyelitis, or other types of encephalitis.These changes suggested that mild regression had occurred since the imaging performed in December 2024.

A total spinal cord MRI (1/16/2025) showed scattered nerve root enhancement in the cauda equina, and Guillain-Barré syndrome (GBS) was suspected. Multiple extensive T2W hyperintense lesions were noted in the cervical and thoracic cords and conus medullaris. Features suggestive of myelopathy suggested GBS-spectrum disease, ADEM, or transverse myelitis. The four-limb nerve conduction velocity test showed normal motor and sensory findings, and the EEG was negative. HBV-related ADEM+/GBS spectrum disease was diagnosed, and intravenous methylprednisolone was continued (2–3 mg/kg/d). IVIG (total 2 g/kg) was re-administered, and levetiracetam combined with lacosamide and entecavir was continued for anticonvulsant and anti-HBV treatment, respectively. The convulsions ceased, and her consciousness improved. She began to recover her speech function after the second IVIG treatment. She was hospitalized for approximately 2 months, and the total disease course was nearly 3 months. On performing cerebral MRI (1/16/2025), the previously noted T2 hyperintense signals in the cortical and subcortical white matter of both cerebral hemispheres had resolved; there were no new lesions observed in the brain or brainstem, and the faint intramedullary T2 hyperintense signals were absent. No abnormal enhancement was observed within the cord, and no cord expansion or atrophy was observed. After discharge, the methylprednisolone was administered orally and gradually tapered, and levetiracetam combined with lacosamide and entecavir was continued. Two months after discharge, the patient's nervous system had completely recovered, the hepatitis B load had decreased, and her liver function remained normal.

## 3 Discussion

To our knowledge, this is the first reported pediatric case of HBV-associated ADEM+/GBS spectrum disease. Cases of HBV infection detected in the CSF are rare. The first step after detecting HBV in the CSF is to determine whether it is due to blood-borne contamination or intracranial HBV replication. In this case, two CSF examinations were performed: HBVDNA was detected in two different CSF samples, hepatitis B surface antigen was positive, and no red blood cells were found in routine CSF testing.

Possible hypotheses regarding how peripheral blood HBV enters the CSF and replicates in the central nervous system (CNS) include blood-brain barrier (BBB) rupture, hepatitis B immune complex production, and virion and/or subviral particle transfer across the BBB (3–6). Pronier et al. reported two cases of HBV infection with neurological lesions, in which high HBVDNA and HBsAg levels were observed in the CSF (5). In this case, no injury occurred during the two lumbar punctures, and no red blood cells were observed in the CSF.

The relationship between HBV replication in the CSF and CNS damage remains inconclusive (7, 8). Liu et al. identified active HBVreplication in a patient with anti-CASPR2 myeloencephalitisand speculated that HBV infection of the CNS might induce autoantibodies against CASPR2, thereby inducing autoimmune encephalitis (9). In this case, anti-GQ1b/anti-GD1b antibodies were also found in the CSF, accompanied by corresponding changes in ADEM/GBS spectrum disorders in the brain and spinal cord, suggesting hepatitis B-related ADEM+/GBS spectrum disease. Hepatitis B is associated with immune-mediated CNS diseases, such as autoimmune encephalitis (9), transverse myelitis (10), ADEM (7), and GBS (4, 11, 12). Few cases have been reported in adults. The mechanisms underlying HBV infection and immune-mediated CNS damage remain unclear. Possible mechanisms for these adverse neurological events include molecular mimicry between HB antigen(s) and myelin proteins or nonspecific activation of autoreactive lymphocytes (7, 13).

CSF may serve as a reservoir for HBV replication (14). No additional treatments are available for HBV replication in the CSF. Cai et al. (3) reported a case of optic neuritis complicated by HBVDNA replication in CSF. Neurological symptoms were alleviated after treatment with atumumab and inebilizumab without hepatitis B recurrence or liver function deterioration. The current patient received entecavir for anti-HBV treatment and systemic glucocorticoids and immunoglobulins to address nervous system damage. She did not develop HBV resistance, and liver function did not deteriorate. During follow-up, the HBV load decreased significantly, liver function remained normal, and the patient fully recovered from the nervous system damage.

## 4 Conclusion

HBV infection is rarely associated with nervous system damage. Early diagnosis, and timely treatment with systemic hormones and immunoglobulin affect the degree of damage to and recovery time of the nervous system in children.

## Data Availability

The raw data supporting the conclusions of this article will be made available by the authors, without undue reservation.
